# Practice makes perfect sense to me: a retrospective analysis of recommending independent skills practice in music therapy for military-connected individuals

**DOI:** 10.3389/fneur.2026.1761700

**Published:** 2026-04-16

**Authors:** John D. Hogue, Clayton Cooke, Melissa Walker Freeman, Rebecca Vaudreuil

**Affiliations:** 1 Henry M. Jackson Foundation for the Advancement of Military Medicine, Inc. in Support of Creative Forces: NEA Military Healing Arts Network, Bethesda, MD, United States; 2 National Intrepid Center of Excellence, Walter Reed National Military Medical Center, Bethesda, MD, United States

**Keywords:** homework, military service members, music therapy, PTSD, symptom reduction, TBI

## Abstract

**Introduction:**

Therapeutic disciplines have researched the use of independent skills practice (i.e., homework) and its impact on patient outcomes, but research on this topic in music therapy has been limited and contradictory.

**Methods:**

This retrospective, exploratory study used a correlational approach to explore the use of recommending independent skills practice between music therapy sessions for military-connected individuals with traumatic brain injury (TBI) and/or post-traumatic stress disorder (PTSD). Board–certified music therapists (MT-BCs) developed a standardized templated note (i.e., a standardized clinical documentation form) that tracked patient care outcomes of military-connected individuals across treatment. Relevant to this paper, the template captured the number of patient-reported symptoms for each session and if the MT-BCs recommended independent skills practices (i.e., homework) for the patient to apply outside of sessions. If MT-BCs recommended independent skills practice, they recorded the goal-based skill the practice addressed.

**Results:**

This paper found that patients who had more sessions wherein MT-BCs recommended independent skills practice reported fewer symptoms both in the following session and at discharge. This paper also provides examples of independent skills practice recommendations and found that 80% were related to either treatment or life conditions. Although this study is retrospective and correlational, it provides preliminary evidence that recommending independent skills practice that is aligned with treatment goals for patients to complete between music therapy sessions could potentially be linked to a more beneficial therapeutic process.

**Discussion:**

This paper discusses purposeful methods for independent skills practice and their impact on music therapy treatment for military-connected populations with TBI and/or PTSD. However, stronger, randomized-controlled trials are needed.

## 1 Introduction

To develop skills that impact their lives and make meaningful changes, Dreier (1) stated that people need to engage in consistent practice across multiple, functional areas of their lives. Patients must connect what they do in therapy to their lives outside of therapy in ways that address their clinical concerns. Thus, therapy should not be an isolated endeavor to garner change; rather, it should coordinate with patients’ lives between sessions. Addressing these changes solely in the therapeutic space could be counterproductive.

Recommending therapeutic activities for patients to complete between sessions is a common, transtheoretical approach across healthcare treatment (2, 3). Surveys of practitioners found that many (83%) practitioners in medicine, psychology, social work, counseling, and nursing reported making therapy-based recommendations (4). Although surveyed therapists using cognitive behavioral therapy (CBT) were more likely to recommend therapeutic activities than other orientations (5), almost all (95–98%) surveyed psychologists made recommendations at least some of the time (4, 5) but in only 57% of sessions (5). Examples of activities could include (a) recording negative automatic thoughts to capture, evaluate, and restructure them for transferability to daily life or (b) targeted exercises to strengthen muscles post-injury. Regardless of the discipline, providers who made recommendations often observed better therapeutic outcomes in their patients (6).

Past research used various terms to investigate this phenomenon including homework, between-session homework or assignments, extra-therapy assignments, *in vivo* behavioral practice, *in vivo* exposure, home practice activities, and action plans (7). Overall, this practice is defined as activities that transfer knowledge from the session into everyday life to address the problems at the source (8). Some clinicians and researchers advised against using the word homework (9) because of its association with education and risk of patients feeling judged (10). Instead, this paper uses the term “independent skills practice” (ISP), because it is consistent with Dreier’s (1) theory, and it better reflects how music therapists help patients generalize treatment to everyday life. The purpose of this exploratory, correlational study was to explore the use of recommending independent skills practice in music therapy (MT) treatment for active-duty service members and veterans (military-connected individuals) with traumatic brain injuries (TBI) and/or post-traumatic stress disorder (PTSD) by analyzing data from a standardized note template (i.e., standardized clinical documentation form).

MT is the clinical and evidence-informed use of music interventions to accomplish non-musical treatment goals through therapeutic alliance with a credentialed professional who has completed a MT degree from an approved academic institution, fulfilled clinical internship training requirements, and passed an exam to earn credentials of board-certified music therapist (MT-BC), which are required for practice (11). MT interventions address various healthcare goals and can provide psychoeducation (12).

Non-MT meta-analyses on distal treatment outcomes showed that ISP positively and causally improved therapeutic outcomes (13) and showed a positive, correlational relationship (14). A review also showed these benefits existed across delivery methods, such as outpatient or intensive residential treatment (15). Patients who engaged in ISP more often had stronger benefits than patients who engaged less often (8, 16), and these benefits appeared from session-to-session (17) and at mid-treatment (16). For TBI specifically, a randomized controlled trial that used ISPs in their protocols found improvements in community functioning (18). Also, one case study realized that the ISP naturally extended the session work into everyday life (19).

Another study integrated music and cognitive behavioral therapy (CBT) and extended in-session work (e.g., singing, playing musical instruments, composing songs using CBT principles) by recommending that patients record their mood and anxiety levels, self-prescribe a song to improve their mood, and identify other factors that improved their mood outside the session. Participants enjoyed the music-based CBT group more than the treatment-as-usual group, and their disability scores decreased, but anxiety and depression scores did not significantly change (20). ISP recommendations through a combination of MT and CBT have also helped children reduce their smart phone and internet addictions, attention-deficit/hyperactivity disorder, and impulsivity scores. Moreover, the children attended more MT and CBT sessions than CBT-only sessions (21).

Using music in everyday life appears to be an essential element of why MT is successful for clients (22). For example, a case study used music in sessions to address empowerment and depression. After a life event, she started singing in a choir outside of sessions when she previously felt too worthless to join. After realizing she needed to apply daring musical combinations in sessions, she applied that logic to her life to combat depression and improve her life (23). Another case study followed a patient through a self-directed ISP of playing the organ outside sessions to reduce anxiety. The patient stated, “...if I hadn’t been able to get hold of that organ that I have at home, I wouldn’t have gained so much from the [MT]” (22), p. 215.

Other qualitative and quantitative studies have found benefits to using ISP. Qualitative studies discussed patients’ benefits from ISP recommendations. Patients reported that ISP recommendations helped relieve anxiety through drumming, connect with friends and family by creating and sharing playlists, and manage auditory hallucinations through listening to music (24). Patients in a mental health unit receiving MT reestablished their connection with music, which promoted their health in their everyday lives, improved their symptoms, and enhanced their quality of life (25). Patients’ moods improved when they completed ISPs of daily music listening for 15–20 min and wrote reflections based on scripts that matched the session’s theme(s) (26). This same design with pre-post quantitative measures found improvements in tension, depression, anger, fatigue, and confusion. ISP was not required, but patients reported that they listened to music for durations of 30 min up to 7 h per week (27).

In a program evaluation, MT-BCs recommended patient-specific musical (e.g., listening to or playing music) and non-musical (e.g., walking or talking) ISP options. The patients stated that ISP helped them improve their depression and emotion regulation techniques (28). One patient even stated that it was simple, that it helped a lot, and that they continued to do it. MT-BCs also recognized that ISP was constructive to treatment through patients’ compliance with the recommendations (29). In another program evaluation, patients participated in group MT, and the MT-BC recommended an ISP of recording positive coping strategies and difficult emotions outside of sessions. The patients improved their depression, which remained low at the four-week follow-up compared to no improvements in control conditions (30).

Even though these studies show benefits with ISP, not all research has shown positive benefits. Researchers in a randomized-controlled trial could not recreate the value of ISP recommendations in MT. They asked some people to engage in music improvisation, which was audio recorded. The researchers recommended patients listen to their recordings at home and keep diaries (31). Although quantitative analyses found that this ISP did not affect depression (32), a follow-up, qualitative analysis found that patients reported positive and negative reactions while also deeply connecting with the music. The majority of Erkkilä et al.’s (32) patients experienced distress and did not comply with the ISP recommendation (33).

Overall, the existing literature evaluating the impact of ISP recommendations on MT treatment outcomes is minimal and often contradictory. For example, Snape et al. (33) found qualitative benefits while Erkkilä et al. (32) did not find quantitative benefits with the same protocol, and some articles reported improvement in depression scores (27, 28, 30) but not others (20, 32). The existing MT research has evaluated ISP’s effect on women with breast cancer recruited from a cancer center (27), people with depression recruited from the general population (32, 33), and students with depression recruited from a university (28, 30); however, it has not yet evaluated the use of ISP with military-connected populations. Further, MT research has not addressed TBI and PTSD symptoms treated in MT that recommends ISP.

However, there is limited, non-MT literature that found beneficial impacts of ISP for veterans with PTSD. One article reported four case studies of veterans who followed an intensive outpatient, cognitive processing therapy program that incorporated ISP worksheets. All four veterans completed the worksheets and three showed reductions in PTSD scores (34). In another article, although 87% of veterans who engaged in at least one ISP recommendation with peer support completed treatment—93% completed treatment with two engagements and 97% completed treatment with three engagements—only 56% of those who did not engage in ISP completed treatment (35).

To further investigate the use of ISP in MT treatment for military-connected individuals with TBI and/or PTSD, the current study conducted an exploratory, retrospective analysis of standardized note template data that tracked military-connected patients’ progress in each MT session. Because existing MT research has not studied the effect of ISP recommendations on treating TBI and PTSD in the military-connected population and due to inconsistent findings on ISP recommendations, this study explored research questions focused on ISP recommendations in the military-connected population, including (a) how often and in what ways the MT-BCs recommended ISP? and (b) does a higher frequency of ISP recommendations relate to a greater reduction in the number of patient-reported symptoms? These research questions aim to deepen the understanding of existing research, which found that more recommendations were related to higher percentages of treatment benefits and completion (35).

## 2 Materials and methods

### 2.1 Participants

Eight MT-BCs and two MT interns used the standardized note template across seven United States (U. S.) Department of War (DoW) clinics that provide rehabilitation for military-connected individuals with TBI and co-occurring disorders, such as PTSD. The MT-BCs and MT interns were part of the Creative Forces®: NEA Military Healing Arts Network (CF), which is an initiative of the National Endowment for the Arts (NEA) in partnership with the US DoW and Department of Veterans Affairs that seeks to improve the health, well-being, and quality of life for military and veteran populations exposed to trauma, as well as their families and caregivers. CF places creative arts therapies at the core of interdisciplinary, patient-centered care at clinical sites throughout the country, includes telehealth services, and increases access to community arts activities (36).

MT is clinically positioned to treat both physiological and psychological health-related concerns. Patients are referred to MT for a specific issue related to a diagnosis, and referral rationale is used to inform goal development, treatment planning, and identify MT interventions that best address patients’ presenting problems that can be physiological and/or psychological. CF has employed standardization efforts for MT interventions commonly facilitated by CF MT-BCs/Interns, which have been adopted broadly across networked sites (37).

The clinics in which MT-BCs/Interns worked typically treat mild traumatic brain injury and related psychological health concerns, as captured in the note template data. Patients enrolled in these clinics are medically stable and engage in treatment as part of continued care for rehabilitation of neurological, psychological, and other health issues. Patients receiving MT primarily experienced mild TBI and were medically stable at the time of MT treatment, thus, they were able to accurately identify and report their symptoms.

The analyzed dataset included data if (a) the patient had at least three MT sessions and (b) the patient was discharged from MT. Within these selected data, the 10 MT-BCs/Interns on average recommended ISP in 54.6% (*SE* = 33%) of sessions per therapist (Min = 0%, Max = 92%). Nine MT-BCs/Interns recommended ISP in at least one session. All MT-BCs held a current board-certification, and the two MT interns were supervised by a MT-BC.

The final selection included 184 unique patients (Age: *M* = 36.41, *SD* = 9.50). Between October 2020 and March 2024, the patients had between three and 33 sessions (*M* = 8.15, *SD* = 4.82) and were seen for an average of 1.88 sessions per month (*SD* = 0.55) across an average of 4.18 months (*SD* = 1.90). On average, 58% (*SD* = 30%) of their sessions included ISP recommendations (Min = 0%, Max = 100%). See Table 1 for additional demographic information. The MT-BCs/Interns administered the majority of sessions in person (96%) vs. telehealth (3%), in longitudinal/outpatient programs (96%) vs. intensive outpatient patient programs (4%), and for 60 min (98.6%) vs. 90 min (0.66%) or 120 min sessions (0.13%). To protect patients’ personally identifiable information, the authors requested demographic frequencies separately from another database, which resulted in receiving missing demographic information in each category.

**Table 1:** Demographics.

Demographic category	Demographic options	Count	% of total
Sex	Men	143	77.72%
	Women	25	13.59%
	Missing Data	16	8.70%
Marital status	Divorced	1	0.54%
	Married	43	23.37%
	Single	10	5.43%
	Missing Data	130	70.65%
Lineage	American Indian or Alaska Native	1	0.54%
	Asian	3	1.63%
	African American	35	19.02%
	Native Hawaiian or Pacific Islander	10	5.43%
	White	99	53.80%
	Other	15	8.15%
	Missing Data	21	11.41%
Branch of Service	Air Force	3	1.63%
	Army	37	20.11%
	Dependent	2	1.09%
	Marines	21	11.41%
	Navy	7	3.80%
	Veteran	1	0.54%
	Space Force	1	0.54%
	Missing Data	112	60.87%
Diagnosis	TBI Only	83	45.11%
	PTSD Only	4	2.17%
	Both	77	41.85%
	Other	20	10.87%

### 2.2 Tools

**Figure 1:**
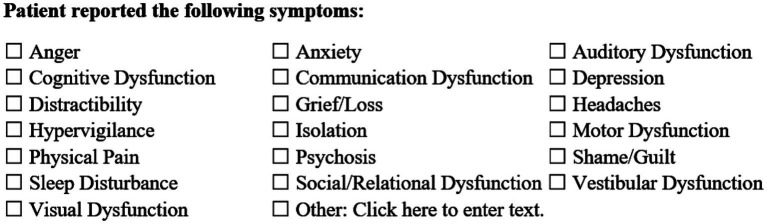
List of standardized options of TBI and PTSD symptoms in the note template.

A team of CF MT-BCs and the healthcare informaticist collaboratively built a note template to standardize documentation across the network for individual MT sessions. The note template used a SOAP-note (i.e., Subjective, Objective, Assessment, Plan) format and included patient-reported symptoms (Figure 1), intervention(s) used in the session (see Table 2 for examples), goals (Table 3) and symptoms (Table 4) that the intervention(s) addressed, and if the MT-BC/Intern recommended ISP. Goals and symptoms were in two separate lists and were usable in any combination. The MT-BCs who built the note template thoroughly discussed what goals and symptoms to include and how they clinically presented, and each goal and symptom included in the note template received consensus from the MT-BC team. If recommending ISP, the MT-BC/intern could describe it through an open text field in the note template.

**Table 2:** Example list of standardized options in the note template for MT interventions.

MT interventions
Active music making—instrumental
Active music making—voice
Assessment
Intentional listening
Lyric analysis/song-discussion
Music technology/production
Nonmusical discussion
Performance
Song writing
Other

**Table 3:** List of standardized options in the note template for addressed goals.

Goals
Attention/concentration/flow
Communication/ openness
Coping/state shifting ability
Emotion regulation
Executive function
Fine motor
Gross motor
Meaning making
Pain management
Positive emotions
Quality of life/engagement/connection
Relationships/relational growth
Self-efficacy in actualizing change
Self-perception/expression
Sense of self/identity development
Sleep hygiene
Social skills/engagement/connection
Other

**Table 4:** List of standardized options in the note template for addressed symptoms.

Symptoms
Anger
Anxiety
Auditory dysfunction
Cognitive dysfunction
Communication dysfunction
Depression
Distractibility
Grief/ loss
Headaches
Hypervigilance
Isolation
Motor dysfunction
Physical pain
Psychosis
Shame/guilt
Sleep disturbance
Social/relational dysfunction
Vestibular dysfunction
Other

An informatics team within the DoW built the note template into TBIPortal within the CarePoint healthcare portal. TBIPortal is a secure application used within the Defense Health Agency that functions as a clinical interface to the TBI patient registry and provides a consolidated view of patient data that informs clinical decision making across the Military Health System in support of patient-centered care (38). TBIPortal provided dynamic entry and allowed the MT-BCs/Interns to enter up to five interventions, as well as five accompanying goals per intervention and five symptoms per goal. The portal securely stored the data and allowed the MT-BCs/Interns to enter the data and download the note, which they then entered into patients’ electronic medical record.

### 2.3 Procedure

After building the standardized note template, the healthcare informaticist trained the MT-BCs/Interns on how to enter unique session data into TBIPortal. The authors obtained IRB approval from Walter Reed National Military Medical Center (protocol: WRNMMC-EDO-2025-1352) and received a de-identified dataset. Three of the authors on this paper were MT-BCs, but only one author treated the patients within this dataset. To maintain anonymity, the author who treated patients included in the dataset never saw the de-identified dataset and only consulted on the group-level analyses to provide context and help with interpretation.

### 2.4 Statistical analyses

A content analysis of 887 ISP recommendation descriptions involved coding them into seven categories: describing (1a) family inclusion or (1b) community engagement; relating to documented (2a) interventions, (2b) addressed goal(s), or (2c) addressed symptom(s) to the ISP recommendation; and (3a) not containing enough information or (3b) not relating to the other categories. Each category received a zero (0) if the text did not describe the category or a one (1) if the text did. One author coded, and another author independently verified the coding.

A paired-sample *t*-test compared the total number of symptoms between initial and discharge sessions, and a between-subjects *t*-test compared the number of symptoms in each session between patients receiving ISP recommendations in the previous session against patients who did not receive ISP recommendations. McNemar tests compared the number of patients reporting each symptom from initial to discharge sessions.

The main analysis used a stepwise, hierarchical, ordinary least squares regression using the number of symptoms in the initial session (step 1), the percentage of sessions with ISP recommendations [step 2, coded as zero (0) for Not Discussed or one (1) for ISP Recommended], and their interaction (step 3) to predict the number of symptoms in the discharge session. A follow-up analysis with PROCESS (39) found the interaction’s simple slopes.

## 3 Results

### 3.1 Content analysis of ISP recommendations

Most (80%) of the descriptions connected to the addressed intervention, goal, or symptom or described family involvement or community engagement in any possible combination. Only 16% of the descriptions did not contain enough information to code, and 4% did not relate to any category. See Table 5 for examples and Table 6 for the correlations among the categories. The correlations showed that when descriptions related to the addressed goal(s), they often also related to the intervention(s) and the addressed symptom(s).

**Table 5:** Examples of ISP recommendation descriptions, documentation information, and coding.

ISP recommendation description	Session intervention(s)	Addressed goal(s)	Addressed symptom(s)	Coding
Incorporating active music playing for 5–15 min on a daily basis and monitoring potential progress in cognitive abilities and anxiety levels	active music making—instrumental	memory	anxiety	intervention(s), goal(s), and symptom(s)
Patient plans on advocating for himself in his work life. Patient wants to work a day or two to help himself get back on a routine and push himself to not self-isolate. Patient will continue writing to externalize his emotions and be able to process through them	songwriting	self-perception/ expression, meaning making		intervention(s), goal(s)
Practicing music relaxation at home, either with family and/or alone, at least 2–3 times by upcoming session, in support of sleep hygiene, coping/state shifting ability, and pain management goals	assessment	communication/ openness, pain management	sleep disturbance	intervention(s), goal(s), symptom(s), and family involvement
Patient will continue to utilize mood playlists and lyric analysis for emotional expression and regulation. Patient will encourage family members to create mood playlists as well	lyric analysis/song-discussion	self-perception/ expression	anxiety	intervention(s), goal(s), symptom(s), and family involvement
Completing assigned task	assessment	communication/ openness		not enough information
Service Member will continue independent play via guitar to promote skills for increased relaxation	non-musical discussion	sense of self/ identity development		not related to the categories

**Table 6:** Correlations among the content analysis categories.

Content analysis coding options	1	2	3	4	5	6	7
1. Intervention(s)	1.00						
2. Addressed Goal(s)	0.15**	1.00					
3. Addressed Symptom(s)	0.08	0.21*	1.00				
4. Family Involvement	−0.04	0.05	−02	1.00			
5. Community Engagement	−0.04	−0.02	−0.03	0.03	1.00		
6. Not Enough Information	−0.62**	−0.34**	−0.19**	−0.08*	−0.09*	1.00	
7. Did Not Relate	−0.29**	−0.16**	−0.12**	−0.04	−0.04	−0.09**	1.00

Bolded and starred values indicate significant relationships, *p < 0.01, **p < 0.05.

### 3.2 Overall symptoms

#### 3.2.1 Total number of symptoms

The total number of symptoms reduced from the initial session (*M* = 4.21, *SE* = 0.23) to the discharge session (*M* = 2.34, *SE* = 0.22), *t*(183) = 9.52, *p* < 0.001, Cohen’s d = 0.70.

#### 3.2.2 Rate of specific symptoms

**Figure 2:**
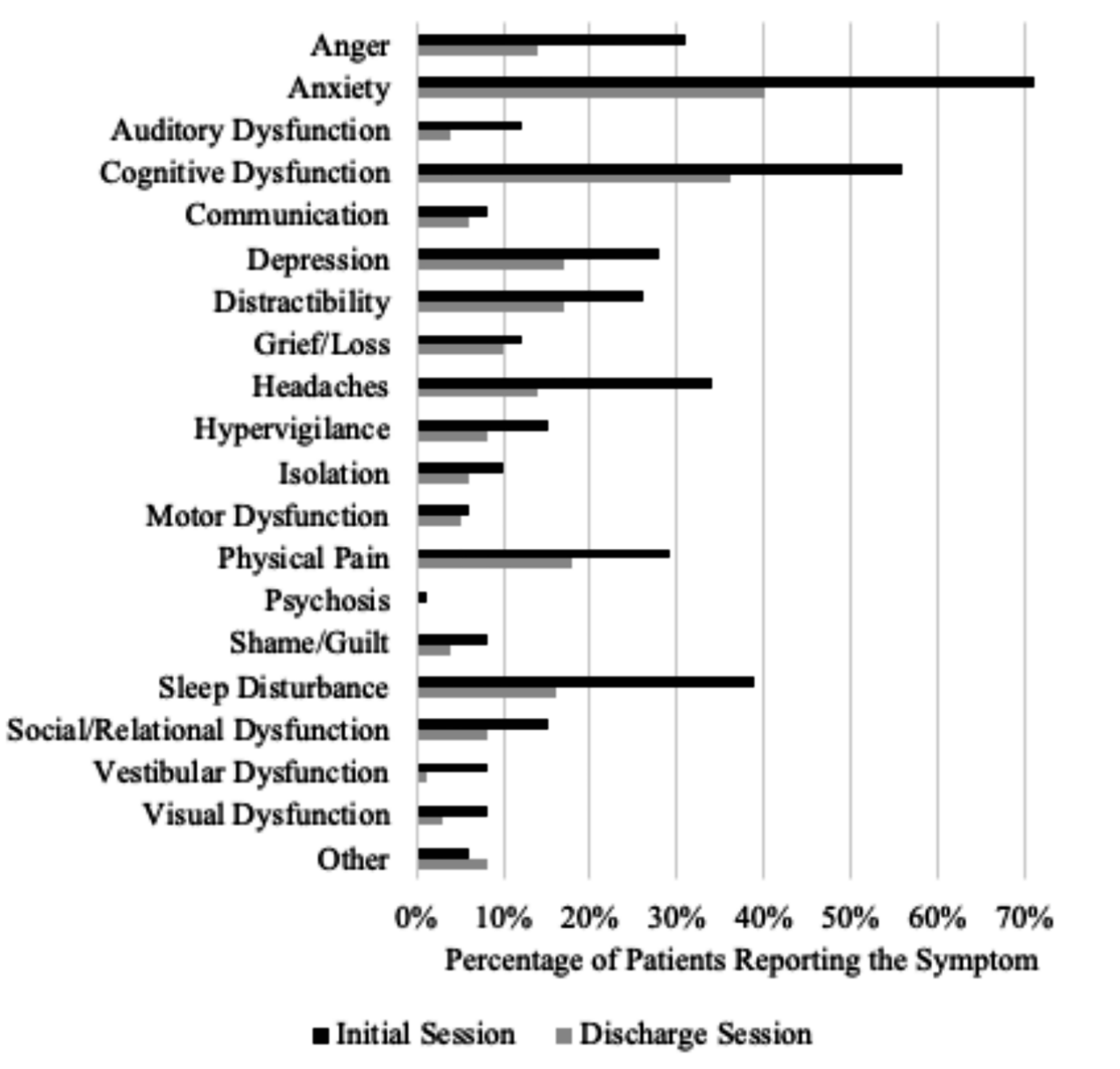
Percentages of patients with the reported symptoms at initial session and discharge.

McNemar tests (Figure 2) revealed fewer patients from initial to discharge session reported anger (*p* < 0.001), anxiety (*p* < 0.001), auditory dysfunction (*p* = 0.001), cognitive dysfunction (*p* < 0.001), depression (*p* < 0.003), distractibility (*p* = 0.010), headaches (*p* < 0.001), hypervigilance (*p* < 001), physical pain (*p* = 0.001), sleep disturbance (*p* < 0.001), social/relational dysfunction (*p* = 0.045), vestibular dysfunction (*p* < 0.001), and visual dysfunction (*p* < 0.001). Analyses did not reveal significant differences for communication (*p* = 0.344), grief/loss (*p* = 0.500), isolation (*p* = 0.118), motor dysfunction (*p* = 0.745), shame/guilt (*p* = 0.090), or other (*p* = 0.543).

### 3.3 Symptoms by previous session recommendation

Patients reported fewer symptoms in the following session if the MT-BC/Intern recommended ISP in the previous session (*M* = 2.38, *SE* = 0.07) than if the MT-BC/Intern had not recommended ISP in the previous session (*M* = 4.35, *SE* = 0.18), *t*(640.77) = 10.31, *p* < 0.001, Cohen’s d = 0.69.

### 3.4 Predicting the number of symptoms in the final session

**Figure 3:**
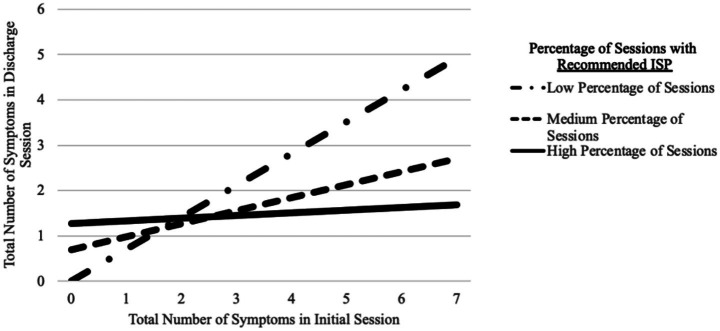
Interaction between the number of symptoms in the initial session and the percentage of sessions with ISP recommendations.

Step 1 revealed that patients had more symptoms in their discharge session if they had more symptoms in their initial session, *R*^2^ = 0.371, B = 0.585, *t*(182) = 10.36, *p* < 0.001. Step 2 revealed that the patients reported fewer symptoms in their discharge when they had a higher percentage of sessions with ISP recommendations (B = −2.80, *t*[181] = −3.73, *p* < 0.001), which significantly explained an additional 4.5% of the variance, Δ*F*(1, 181) = 13.90, *p* < 0.001. The interaction in Step 3 was also statistically significant (B = −1.100, *t*[180] = −7.78, *p* < 0.001) and significantly explained an additional 14.7% of the variance, Δ*F*(1, 180) = 60.51*. p* < 0.001. PROCESS was used to determine simple slopes. For Patients with low (b = 0.745, *t*[180] = 13.13, *p* < 0.001) and medium (b = 0.287, *t*[180] = 4.94, *p* < 0.001) percentages of sessions with ISP recommendations, the number of symptoms in the discharge session increased as they had more symptoms in the initial session. However, patients with a high percentage of sessions with ISP recommendations remained at a consistently low number of symptoms in the discharge session regardless of the number of symptoms in the initial session, b = 0.058, *t*(180) = 0.746, *p* = 0.456. For patients with three or more symptoms in the initial session, the number of symptoms in the discharge session was lowest for patients with a high percentage of sessions with ISP recommendations. See Figure 3 for the interaction and Table 7 for correlations.

**Table 7:** Correlations.

Regression analysis variables	1	2	3
1. Initial Session Total Symptoms	1.00		
2. Discharge Session Total Symptoms	0.61**	1.00	
3. Percentage of Sessions with ISP Recommendations	−0.28**	−0.37**	1.00

Bolded and starred values indicate significant relationships, **p < 0.01.

## 4 Discussion

This exploratory, retrospective study sought to evaluate ISP recommendations on MT treatment outcomes for military-connected individuals with TBI and/or PTSD. Although this was a correlational study without the proper controls to determine causality (see the limitations below), it provided preliminary and foundational evidence to discuss the potential benefit that ISP recommendations could have on the therapeutic process. Overall, the total number of symptoms significantly decreased from the initial to the discharge session. For 12 out of 18 symptoms included in the note template, the number of patients reporting those symptoms significantly reduced from initial to discharge sessions. However, without a control group or the ability to control for patients who received other types of therapies at the same time they received MT treatment, it is impossible to know if these decreases happened naturally over time or as part of working in tandem with other therapeutic services.

This study also found that symptoms were lowest at discharge when the MT-BC/Intern recommended more ISPs across treatment than when they offered fewer or no ISPs. Patients who received ISP recommendations in the previous session had fewer symptoms in the next session than patients without ISP recommendations, and patients with a high percentage of sessions with ISP recommendations and a high number of symptoms in the initial session had a lower number of symptoms at discharge than patients with fewer ISP recommendations and higher symptoms in the initial session. These findings are similar to Hernandez-Tejada et al. (35), who found that more ISP recommendations were associated with higher program completion rates, and they provide preliminary, foundational evidence demonstrating that ISP recommendations could play an important part in the therapeutic process for MT-BCs/Interns treating the military-connected populations.

Also, the content analysis showed that 80% of the ISP recommendations connected to either the addressed goal or symptom, implemented intervention, or mentioned community and/or family engagement. These connections indicate that MT-BCs**/**Interns related ISP recommendations to the treatment process and/or the patients’ lives, which may have been a factor in the lower number of symptoms in the discharge session. Erkkilä et al. did not connect ISP recommendations to either the sessions’ theoretical orientation, the MT-BCs’ expertise, or the patients’ self-directed interests (33), but Bong et al. (21) and the other program evaluation (28–30) and case study articles did (22, 23). Only one MT-BC in the Erkkilä et al. protocol discussed ISP in every session, and others either forgot or chose not to discuss it due to a conflict between the protocol’s structured approach and their theoretical orientation. Moreover, those patients experienced technological problems with playing the music, diminished motivation, lack of time, high amounts of stress, and increased depression and anxiety (33). In the current study, almost all (90%) MT-BCs/Interns recommended ISP in at least one session, and the content analysis of the ISP recommendations showed that MT-BCs/Interns related the recommendations to treatment (e.g., addressed goals, addressed symptoms, and/or interventions). Therefore, it is likely that patients in the current study received more ISP recommendations than in the Erkkilä et al. protocol, and that the MT-BCs/Interns in the current study designed them to be purposeful, patient-specific, and related to the therapeutic process.

The importance of purposeful ISP recommendations is clearly stated through research in non-MT therapeutic disciplines. Connecting ISP recommendations to in-session work, such as the interventions, goals, and symptoms, is a foundational requirement for ISP recommendations (8, 40). Ryum et al. (41) argued that therapists should make recommendations based on the therapist’s therapeutic approach, their collaborative discussions with the patient, and if the ISP recommendations are natural extensions of the work done in session.

This study found a diverse range of ISP recommendations (see Table 4 for examples), which is similar to the past research (8, 42, 43), and it suggests that the MT-BCs/Interns in this study collaborated with patients to co-construct recommendations, supporting Ryum et al. (7) who stated that this is a priority. The results of this study imply that the MT-BCs/Interns recommended ISP at similar rates compared to other disciplines, where 90% of the MT-BCs/Interns made an ISP recommendation at least once, which is similar to more than 80% of healthcare providers making ISP recommendations (4, 5). Also, MT-BCs/Interns made ISP recommendations in 58% of sessions in the current study compared to 57% of sessions in the past non-MT research (5). These similarities suggest that MT-BCs can practice therapy similarly to other clinicians. As other therapeutic disciplines may misunderstand MT (44), these results help show MT within a similar context as other therapeutic disciplines.

To our knowledge, this study is the first to evaluate ISP recommendations in MT treatment for military-connected individuals with TBI and/or PTSD and is one of a few quantitative studies within MT [see (20, 27, 28, 30, 32)]. Results from this study and prior studies provided suggestive evidence that ISP recommendations could have therapeutic benefits (13) and indicates using patient-specific recommendations directly related to the therapeutic process as a best practice of providing therapy. Patient-specific ISP recommendations could potentially be important to the therapeutic process for several reasons: (a) patients could practice goal-oriented skills through music more consistently; (b) patients may be more motivated to engage in ISP if they helped co-construct the recommendations or see ISP as helpful (45); (c) patients could perceive that the MT-BCs took an interest in their treatment; (d) patients could assume an active role in their treatment; (e) patients could implement the ISP in more areas of their lives, supporting Dreier (1); and (e) patients could connect benefits from sessions to a community-based experience.

Although there are a plethora of nonmusical community-based experiences that therapists can recommend (46, 47), offering MT ISP recommendations could provide military-connected patients with familiarity, accountability, and/or serve as a form of self-assessment to understand their strengths, needs, and treatment. Familiar contexts within ISP recommendations could provide patients with autonomy and control over their health and lives through enjoyable activities in different settings (48). Where military personnel come to expect regimented environments including healthcare, ISP recommendations could help bridge gaps between military service and civilian life and garner long-term benefits post-therapy by providing treatment through a clinic-to-community continuum (C2CC).

ISP recommendations within the C2CC can be active/expressive (e.g., performing at an open mic night to address anxiety, joining a band to address communication, making music with family to address familial/social issues) or passive/receptive in nature (e.g., attending a concert to address hypervigilance) (49). MT programs (50) and CF (51) help patients meet their needs by implementing arts-based C2CC approaches that span the spectrum from engaging in creative arts therapies, to community arts engagement, and to independent arts practices. C2CC integration warrants that sessions focus on clinical goals, and that patients explore arts experiences that support shifting from creative arts therapies to community arts engagement (51). C2CC-based treatment models can help promote well-being (52), agency (53), social skills, self-esteem, and emotion regulation (54).

For example, a U. S. Navy Corpsman detailed their experience with community integration as part of MT treatment (55), p. 205–206:

The more I learned and got involved with music therapy, the more I realized how much it was helping me. The music therapist would assign [ISP] and that’s how my family was able to get involved; they even attended some [MT] sessions with me. I can honestly say that the shared love of music helped strengthen our family. The more involved I became with [MT], the better I was feeling about many aspects of my life. I started playing bass guitar again and was inspired to explore performance. To further this goal I joined [a band], through which I met other service members and veterans who played music and performed as a team, playing gigs in the community.

This testimonial suggests that the combination of music-based ISP aligned with clinical MT treatment goals provided additional structure that allowed the patient to focus their thoughts and align efforts to create something positive and non-threatening, expand to other functional areas their life, and make meaningful and beneficial changes.

Due to the retrospective, correlational nature of these data collected through routine clinical care, this study has several limitations that are important to acknowledge. As previously stated, these data could not control for natural healing over time or the impact of interdisciplinary therapeutic treatment. Also, as patients progressed in treatment, they may have become increasingly aware of the symptoms they were experiencing and their root causes. Hence, reported symptoms could have increased with the development of higher level of awareness. However, this study found that reported symptoms decreased overall and were lowest when MT-BCs recommended more ISP throughout treatment.

The MT-BCs/Interns in this study did not use a standardized approach to treatment or specific MT interventions; therefore, variations in practice, the therapeutic relationship, and documentation likely existed, despite using a standardized template. Additionally, this study did not use a validated measure, so directly interpreting and comparing results directly with other research is impossible. Each ISP recommendation was targeted to meet each patient’s specific clinical needs; therefore, a formal quality assurance check was not completed. Because of these limitations, further studies should determine causality through randomized, controlled trials and quality assurance checks. The trials could evaluate ISP frequency and duration to determine any additive effects. Future studies could also compare intentional use of patient-specific ISP against a standard assignment.

Furthermore, this study did not track if patients adhered to ISP recommendations. Past research found better outcomes in patients who complied with the therapists’ ISP recommendations (6, 10, 13, 14) and in patients who consistently engaged in ISP (8, 16, 45). Although outcomes have depended on moderations between ISP completion, timing, and type (14, 45), completing and finding merit in ISP also depended on other factors. For example, veterans produced higher quality ISP with brief family involvement and completed marginally more ISP than without familial involvement (56). Future MT research should evaluate ISP engagement types (e.g., individual, collective) and ISP’s impact on outcomes before addressing recent non-MT research topics, such as the effects of complying with ISP at the start of therapy compared to later compliance on therapeutic benefits (57).

Regarding factors related to interdisciplinary care, especially in the intensive outpatient treatment programs, patients could have also received speech-language pathology, physical therapy, occupational therapy, and/or behavioral health in co-treatment sessions with MT or in addition to MT, depending on the site. The note template did not track co-treatments or if the patients received other therapies, but the results could have been influenced by their presence. Future prospective studies could isolate MT treatment when comparing ISP against standard treatment.

Despite the correlational nature of the data and the limitations, this retrospective, exploratory study provided preliminary evidence that ISP recommendations could be an important component of the MT process when treating military-connected individuals with TBI and/or PTSD. It demonstrated the relationships between the variables in a real-world, therapeutic setting, and it suggests that patients with TBI could benefit from purposeful, treatment-oriented ISP compared to standard protocols, which is consistent with a systematic review indicating that patients with TBI need patient-centered treatment (58). These results also provide a foundation for future studies to evaluate causality in ISP engagement.

## Data Availability

The data analyzed in this study is subject to the following licenses/restrictions: we do not have permissions to release the dataset. Requests to access these datasets should be directed to John D. Hogue, hoguemt@gmail.com.

## Author disclaimer

The views expressed in this abstract are those of the authors and do not necessarily reflect the official policy of the Department of War or the US Government. The opinions contained herein represent the private views of the authors and are not to be construed as official or as reflecting the views, opinions, or policies of the Henry M. Jackson Foundation for the Advancement of Military Medicine, Inc., the National Endowment for the Arts, or the US Government. Mention of trade names, commercial products, or organizations does not imply endorsement by the US Government. This material was created free of branding or market affiliations. The authors are operating solely as contributors.
